# A high-throughput assay identifies molecules with antimicrobial activity against persister cells

**DOI:** 10.1099/jmm.0.001856

**Published:** 2024-07-12

**Authors:** Maiken Engelbrecht Petersen, Liva Kjær Hansen, Alexander Alexandrovich Mitkin, Nicholas M. Kelly, Thomas Keith Wood, Nis Pedersen Jørgensen, Lars Jørgen Østergaard, Rikke Louise Meyer

**Affiliations:** 1 Interdisciplinary Nanoscience Centre (iNANO), Aarhus University, 8000 Aarhus C, Denmark; 2 Pincer Biotech ApS, 8000 Aarhus C, Denmark; 3 Department of Chemical Engineering, Pennsylvania State University, University Park, USA; 4 Department of Clinical Medicine, Aarhus University, 8200 Aarhus N, Denmark; 5 Department of Infectious Diseases, Aarhus University Hospital, 8200 Aarhus N, Denmark; 6 Department of Biology, Aarhus University, 8000 Aarhus C, Denmark

**Keywords:** drug development, persister cells, *Staphylococcus aureus*

## Abstract

**Introduction.** Persister cells are transiently non-growing antibiotic-tolerant bacteria that cause infection relapse, and there is no effective antibiotic therapy to tackle these infections.

**Gap statement.** High-throughput assays in drug discovery are biased towards detecting drugs that inhibit bacterial growth rather than killing non-growing bacteria. A new and simple assay to discover such drugs is needed.

**Aim.** This study aims to develop a simple and high-throughput assay to identify compounds with antimicrobial activity against persister cells and use it to identify molecular motifs with such activity.

**Methodology.** We quantified *Staphylococcus aureus* persister cells by enumeration of colony forming units after 24 h ciprofloxacin treatment. We first quantified how the cell concentration, antibiotic concentration, growth phase and presence/absence of nutrients during antibiotic exposure affected the fraction of persister cells in a population. After optimizing these parameters, we screened the antimicrobial activity of compound fragments to identify molecular structures that have activity against persister cells.

**Results.** Exponential- and stationary-phase cultures transferred to nutrient-rich media displayed a bi-phasic time-kill curve and contained 0.001–0.07% persister cells. A short rifampicin treatment resulted in 100% persister cells for 7 h, after which cells resumed activity and became susceptible. Stationary-phase cultures displayed a low but constant death rate but ultimately resulted in similarly low survival rates as the exponential-phase cultures after 24 h ciprofloxacin treatment. The persister phenotype was only maintained in most of the population for 24 h if cells were transferred to a carbon-free minimal medium before exposure to ciprofloxacin. Keeping cells starved enabled the generation of high concentrations of *S. aureus* cells that tolerate 50× MIC ciprofloxacin, and we used this protocol for rapid screening for biocidal antibiotics. We identified seven compounds from four structural clusters with activity against antibiotic-tolerant *S. aureus*. Two compounds were moderately cytotoxic, and the rest were highly cytotoxic.

**Conclusion.** Transferring a stationary-phase culture to a carbon-free minimal medium for antimicrobial testing is a simple strategy for high-throughput screening for new antibiotics that kill persister cells. We identified molecule fragments with such activity, but further screening is needed to identify motifs with lower general cytotoxicity.

## Introduction

Antibiotics sometimes fail to cure bacterial infections – not because the bacteria are resistant to the drug but because they temporarily assume a non-growing state where they tolerate the bactericidal antibiotics available today. The infection therefore becomes persistent, and the bacteria displaying this phenotype are called persister cells. Gladys Hobby first discovered bacterial persister cells in 1942 by observing a small percentage of surviving bacteria after treating a culture of cocci with penicillin [1]. She further observed that these survivors were non-growing. Two years later, Joseph Bigger named this tolerant sub-population ‘persister cells’, and he discovered that the survivors were indistinguishable from the original culture [2]. He hypothesized that these persister cells were no more resistant to penicillin than their non-persister cell counterparts, but instead, they were tolerant of the action of penicillin. Today, persistence is defined as the ability of a subset of a bacterial population to survive exposure to a bactericidal drug concentration although they cannot replicate in the presence of the drug [3]. The presence of persister cells in a population is characterized by a bi-phasic time-kill curve, where susceptible cells are killed at an initial fast rate, leaving behind a population of persister cells that tolerate the antibiotic and are killed at a much slower rate [3].

Persister cells are non-growing and have activated a suite of protective mechanisms that allow them to temporarily cope with starvation or different forms of stress. These include cell envelope stress [4], starvation [5], oxidative stress [6,7], heat stress, DNA damage and the accumulation of misfolded proteins [8]. By arresting growth, slowing metabolism and activating protective mechanisms, persister cells tolerate extremely high concentrations of antibiotics because antibiotics typically target processes involved in growth or require a certain level of metabolic activity to exert their antimicrobial effect [9,11]. Furthermore, it has been hypothesized that the molecular mechanism of persister formation is stress-specific and that there is not one global mechanism [12]. The persister phenotype is often associated with bacterial biofilms, where persister cells exist as a sub-population. Inside the biofilm, bacteria are protected from the immune system, and the non-growing persister cells are thus protected from elimination by professional phagocytes. The persister phenotype is reversible, and bacteria resume growth when conditions are favourable. Therefore, the persister phenotype is often associated with recalcitrant and relapsing bacterial infections [13,14], and there is an urgent need for antibiotics that not only target actively growing bacteria but also persister cells.

Efforts to discover novel antibiotics often rely on high-throughput assays to screen large compound libraries for antimicrobial activity. Standard assays detect antimicrobial activity based on inhibition of bacterial growth in a rich laboratory medium [15,17]. However, this approach is biased towards identifying compounds that target actively growing bacteria and is therefore likely to identify new compounds with the same shortcomings as currently available antibiotics. Antibiotics that target persister cells must kill bacteria independent of their metabolic state. High-throughput screening assays designed for the discovery of anti-persister drugs must therefore measure the loss of viable cells from a highly concentrated population of persister cells.

The first challenge for designing an anti-persister drug screening assay is to reproducibly generate a high concentration of persister cells. Ideally, assays that screen for anti-persister drugs should detect a 1000-fold (i.e. log3) reduction in colony forming units (CFU) to verify biocidal activity according to the ISO standard 20776-1. This seemingly simple requirement is difficult to meet because persister cells comprise a small and variable fraction of bacterial populations. Experimentally, persister cells have been quantified by measuring surviving cells by CFU enumeration after challenging a population with high concentrations of antibiotics (≥10× MIC) in a laboratory growth medium for some hours. The incubation time must be long enough to kill susceptible cells, such that viable cells are enumerated after the bi-phasic time-kill curve has reached the second phase, with the slow death rate representing persister cells [18]. Many studies have estimated the fraction of persister cells in a stationary phase broth culture after 3–5 h incubation with the antibiotic, and the number varies from 0.000001% to just under 1% [18,21]. After such a short incubation time, the susceptible population may not be completely eliminated (i.e. the population is still on the steep part of the time-kill curve), which results in highly variable and time-sensitive results [22]. A reproducible assay for identifying anti-persister drugs should therefore involve much longer incubation times, e.g. 24 h, which is standard in other antimicrobial assays.

The media composition, nutrient concentration and exposure to different stress factors affect how many cells in a population will transition to the persister state. Furthermore, these and other unknown factors affect how quickly the bacteria resuscitate and become susceptible to antibiotics after transfer to fresh growth media. Since the persister fraction is quantified as the population of cells that survive exposure to antibiotics after a specified amount of time, the dynamics of resuscitation will also impact the fraction of viable cells present at the time of sampling. While the death rate of the susceptible population in the first phase of the time-kill curve reflects how fast the antibiotic acts on the cells, it has been shown that the death rate in the second phase does not reflect the action of the antibiotic on non-dividing cells but rather reflects the rate at which a population of inactive cells resumes activity and becomes susceptible [23]. It is thus difficult to discern if a drug is truly active against bacteria in the persister state when testing the efficacy of the drug in a nutrient-rich medium where cells can resume activity.

Much research on persister cells has been performed on *Escherichia coli* [20,23,25], and Kwan *et al*. reported that *E. coli* persister formation could be triggered by treating exponential-phase cultures briefly with rifampicin, tetracycline or carbonyl cyanide *m*-chlorophenylhydrazone (CCCP) to halt transcription, translation or ATP synthesis, respectively. This approach resulted in persister fractions of 10–100 % in *E. coli* [20] as assessed by their tolerance to ciprofloxacin and ampicillin for 3 h. In another study, the pre-treatment with rifampicin initially killed 66 % of the population before turning almost all the remaining cells into highly tolerant persister cells [24], as shown by their tolerance to ampicillin for 2 h. However, protocols for generating persister cells are less well characterized in staphylococci, which are the culprits of many chronic biofilm-associated infections [26]. For *Staphylococcus aureus*, one study showed that CCCP exposure induced the persister phenotype in 60% of the population, as shown by their tolerance to levofloxacin for 3 h [27], indicating that ATP levels also play a role for Gram-positive bacteria. The mechanism for persister formation presumably varies between different bacterial species, particularly between Gram-positive and Gram-negative bacteria. Knowledge is therefore not necessarily transferable between bacterial strains and growth conditions, and protocols for the preparation of antibiotic-tolerant persister cells must be validated for each experimental setting.

The aim of this study was to establish a high-throughput screening protocol to identify small molecules with antimicrobial activity against *S. aureus* persister cells. Our objectives in designing the assay were (1) to generate a high concentration of persister cells that enables detection of a 1000-fold (log3) reduction in viable cells during drug exposure and (2) to expose the bacteria to a drug for 24 h while keeping the cells in the antibiotic-tolerant non-dividing state.

We characterize how the experimental conditions for antibiotic exposure affect the fraction of cells that tolerate antibiotics and can be perceived as persister cells. These experimental conditions include the bacterial cell concentration, the antibiotic concentration, the exposure time and the presence of nutrients during antibiotic exposure. After optimization of the protocol, we screened 250 molecular fragments and identified a cluster with a common structural motif that was active against persister cells. The development of this motif to improve activity and physiochemical properties was then undertaken.

## Methods

### Bacterial strains and growth conditions

The clinical isolate *S. aureus* SAU060112 (DSM 110939) and the type strain *E. coli* K12 (DSM 498) were cultivated on tryptic soy agar (TSA) and in tryptic soy broth (TSB) and grown overnight at 37 °C and 180 r.p.m., diluted 1:1000 in TSB and incubated overnight again before each experiment. Modified M9 (mM9) was used under starvation conditions. mM9 contains 1× M9 salts, 2 mM MgSO_4_, 0.1 mM CaCl_2_, 1 mM thiamine-HCl, 0.05 mM nicotinamide and trace metals [5, 28]. Chemicals were purchased from Sigma-Aldrich unless otherwise stated.

### Minimum inhibitory concentration

The MIC of ciprofloxacin and mitomycin C was determined using broth dilution. Briefly, an overnight culture of *S. aureus* or *E. coli* (5×10^5^ CFU ml^−1^) was added to twofold serially diluted antibiotics in TSB in 96-well plates and incubated overnight at 37 °C and 50 r.p.m. MIC was determined as the minimum concentration where growth was inhibited, as measured by OD_600_. The MIC of ciprofloxacin against *S. aureus* and *E. coli* was 1 and 0.008 µg ml^−1^, respectively. The MIC of mitomycin C against *S. aureus* was 0.8 µg ml^−1^.

### Time-kill curves of exponential and stationary cultures treated with ciprofloxacin or mitomycin C

For exponential-phase cultures, overnight cultures were diluted 1:100 and incubated until reaching OD_600_ 0.1–0.5. For stationary-phase cultures, overnight cultures were diluted to OD_600_ 1 in mM9 or TSB. Cultures were then washed by centrifugation at 13150 ×g for 10 min, resuspended in TSB or mM9, diluted tenfold into TSB or mM9 with 10×, 50× and 100× MIC ciprofloxacin or 10× MIC mitomycin C and incubated at 37 °C at 50 r.p.m.

The CFU was determined at *T*_0_ (prior to mixing with antibiotics) and after 3, 5 and 24 h; 100 µl sample was washed by adding 900 µl mM9, centrifuging cells at 13150 ×g for 10 min and resuspending the pellet in 100 µl mM9. CFU was then determined by tenfold dilution and transferring 10 µl (*S. aureus*) or 100 µl (*E. coli*) to TSA.

### Rifampicin pre-treatment to induce persistence

Overnight cultures of *S. aureus* or *E. coli* were diluted to OD_600_ 1 in mM9 or TSB, washed by centrifugation as described above, resuspended in mM9 or TSB containing 100 µg ml^−1^ rifampicin and incubated for 30 min at 37 °C, 180 r.p.m. Subsequently, the rifampicin-treated samples were washed by centrifugation and transferred to TSB or mM9 with ciprofloxacin as described above.

### Compound screening

For screening, 250 fragment compounds were purchased from Key Organics (Camelford, Cornwall) fragment library, resulting in a cluster of four fragments (compounds 241, 242, 243 and 244) having anti-persister activity and the same 3,5-disubstituted phenol motif. Compounds that incorporated this or a similar motif were purchased from Enamine Ltd. (Kyiv, Ukraine). Compounds #258, 260, 267, 280, 292, 296 and 322 correspond to Enamine compound numbers Z2583036198, Z2668835953, Z2177044396, Z2683009586, Z1669437412, Z2683009578 and Z4562301868, respectively. Compounds were stored at room temperature until dissolved in DMSO (200 mM) and then stored at −20 °C. Overnight cultures of *S. aureus* were diluted to OD_600_ 1 in mM9, washed by centrifugation as described above and resuspended in an equal volume of mM9. Compounds (1, 5 and 10 mM) and bacteria (5×10^7^ CFU ml^−1^) were mixed and incubated at 37 °C, 50 r.p.m. for 24 h, and 10 µl was then spotted on TSA and incubated overnight at 37 °C. CFU was not enumerated, but a rough assessment was made based on the following: >10^4^ CFU ml^−1^ resulted in confluent growth, 10^2^–10^4^ CFU ml^−1^ resulted in distinct colonies and <10^2^ CFU ml^−1^ resulted in no growth.

### Cytotoxicity test

Chinese Hamster Ovarian (CHO) B11 cells (passage number: 13–16) were defrosted and transferred to 37 °C Ham’s F-12 Nutrient Mix (Thermo Scientific, 11765054) and subsequently incubated at 37 °C, 5% CO_2_ and 85% humidity. After 2–3 days of incubation, cells reached confluence and were split using 0.25% trypsin-EDTA (Thermo Scientific, 25200072) to release the cells from the surface. Following 2–3 days of incubation at 37 °C, 5 % CO_2_ and 85 % humidity, cells were split again and incubated using similar incubation conditions. After reaching confluence after 2–3 days, the cell culture was diluted to 10^5^ cells ml^−1^ in Ham’s F-12 Nutrient Mix and seeded in a 96-well plate (Fisher Scientific, 10567131) followed by incubation at 37 °C, 5% CO_2_ and 85% humidity. When reaching 70–90% confluence, cells were treated with compounds 258 (1, 5 and 10 mM), 260 (5 and 10 mM), 267 (1 mM), 280 (1, 5 and 10 mM), 292 (5 and 10 mM), 296 (1, 5 and 10 mM) and 322 (1, 5 and 10 mM) in Ham’s F-12 Nutrient Mix or treated with 0.5, 2.5 or 5% DMSO in Ham’s F-12 Nutrient Mix (solvent controls). The samples were incubated at 37 °C, 5 % CO_2_ and 85 % humidity for 24 h before adding 1× PrestoBlue Cell Viability Reagent (Invitrogen, A13261) and incubating for 2 h. After incubation, the samples were analysed in a CLARIOstar Microplate Reader (BMG Labtech) with excitation at 560 nm and emission at 590 nm.

### Statistical analyses

Data were tested for normality using a Shapiro–Wilk test. Normally distributed data were tested using a *t*-test for single comparisons, a one-way ANOVA using mixed-effects analysis for time-kill curves and ordinary ANOVA for bar graphs and death rates. Equal variability was not assumed, and the Geisser–Greenhouse correction was used. A post-hoc uncorrected Fisher’s least significant difference was performed for one-way ANOVA tests. Non-normally distributed data were tested using a Mann–Whitney *U*-test, and for matched comparisons, a Wilcoxon matched-pairs signed-rank test was run. For normally distributed data, the mean ± standard deviation is shown, whereas for non-normally distributed data, the median ± (range) is shown. GraphPad Prism was used for all statistical analyses [v. 9.5.1 (733) for Windows, GraphPad Software, San Diego, California USA, www.graphpad.com].

## Results and discussion

In this study, we used ciprofloxacin, a fluoroquinolone antibiotic targeting DNA gyrase, as the standard antibiotic to benchmark the effect of novel compounds against persister cells. Mitomycin C was included as a positive control due to its well-documented antimicrobial effect on bacterial persister cells [29]. The MIC values for these antibiotics against *S. aureus* are written in the Methods section.

We first determined whether the concentration of bacteria and antibiotics affected the fraction of viable cells remaining after antibiotic treatment, which we defined as persister cells. These parameters are important to standardize, as the antimicrobial efficacy of antibiotics can depend on the cell concentration (i.e. the inoculum effect) [30,31] and on the antibiotic concentration because the antibiotic-induced activation of prophages at low concentrations can be mistaken for the direct action of the antibiotic [32]. Prophages are common in the genomes of clinical isolates of *S. aureus* and are also present in SAU060112 [33].

**Fig. 1.**
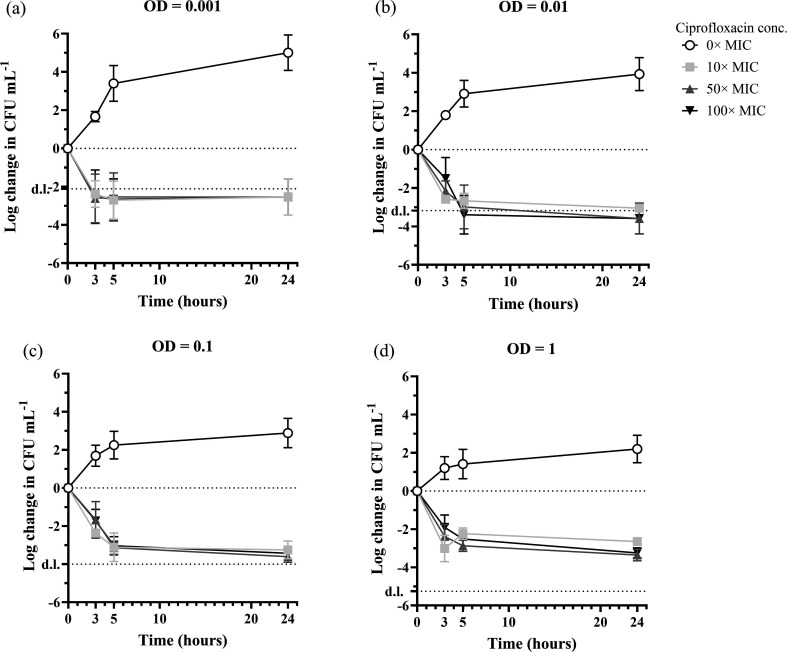
The effect of ciprofloxacin concentration and bacterial concentration on time-kill curves and the fraction of persister cells. Exponential-phase cultures of *S. aureus* were washed and resuspended in TSB at the following optical densities: 0.001 (approximately 5×10^5^ CFU ml^−1^) (a), 0.01 (approximately 5×10^6^ CFU ml^−1^) (b), 0.1 (approximately 5×10^7^ CFU ml^−1^) (c) and 1 (approximately 5×10^8^ CFU ml^−1^) (d) in TSB before being treated with different concentrations of ciprofloxacin at time = 0 h. At 0, 3, 5 and 24 h, viable cells were quantified as CFU *n* ≥ 3 biological replicates. d.l.: detection limit.

A time-kill assay was performed in TSB using four different turbidities (0.001, 0.01, 0.1 and 1) of exponential-phase *S. aureus* cultures (Fig. 1). We quantified CFU after 3, 5 and 24 h, as this is the antibiotic exposure time most commonly used to quantify persister cells [20,27, 34]. At turbidities 0.001 and 0.01, the cell concentration quickly decreased below the detection limit (Fig. 1a and b), and the fraction of surviving cells could therefore not be determined. At turbidities 0.1 and 1, we measured a 3-log reduction in response to ciprofloxacin. Therefore, we decided to use the turbidity of 0.1 (10^7^ CFU ml^−1^) as a starting concentration for the remainder of the study.

### Rifampicin pre-treatment induced persister phenotype in *S. aureus* for at least 7 h

**Fig. 2.**
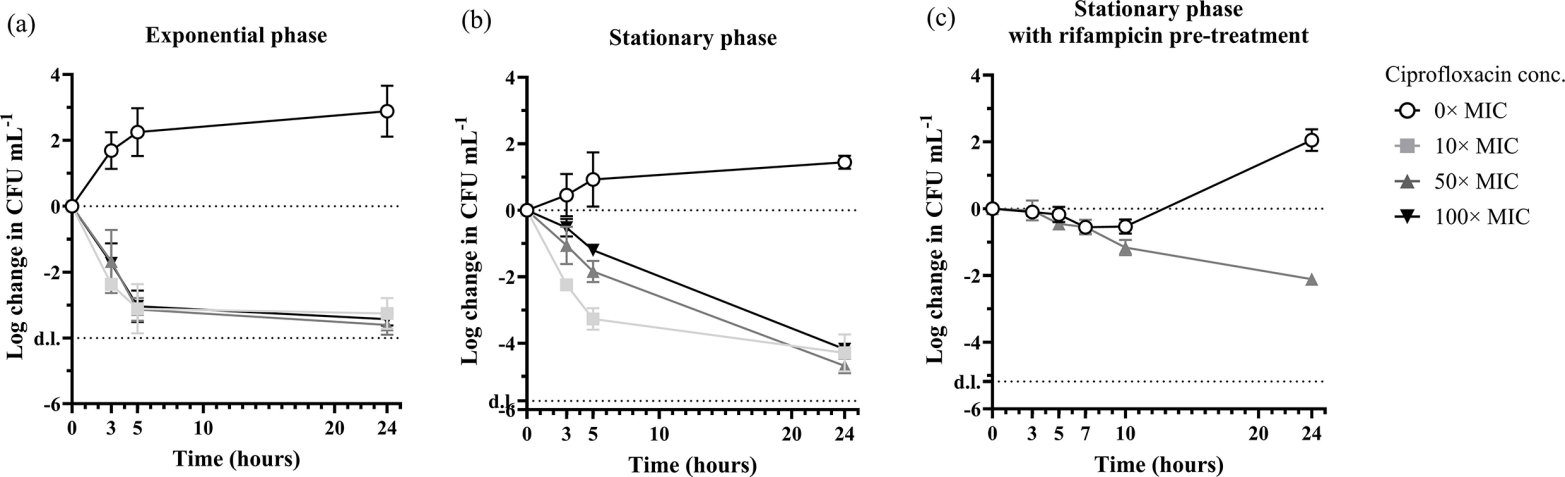
Time-kill curves of *S. aureus* treated with ciprofloxacin in TSB. An exponential-phase culture (approximately 10^7^ CFU ml^−1^) (a) or a stationary-phase culture (approximately 5×10^7^ CFU ml^−1^) (b) was transferred to fresh TSB with or without ciprofloxacin at T = 0 h (1× MIC = 1 µg ml^−1^). (c) A stationary-phase culture of *S. aureus* (approximately 5×10^7^ CFU ml^−1^) was transferred to fresh TSB with 100 µg ml^−1^ rifampicin for 30 min and subsequently washed and transferred to fresh TSB with or without ciprofloxacin at 0 h. d.l.: detection limit, *n* ≥ 3 biological replicates.

The fraction of persister cells is highly dependent on their metabolic state, and we therefore compared the fraction of persister cells in exponential- and stationary-phase cultures, which were exposed to 10×, 50× or 100× MIC of ciprofloxacin for 3, 5 or 24 h. Exponential-phase cultures displayed the characteristic biphasic time-kill curve (Fig. 2a) and plateaued after 5 h treatment (one-way ANOVA, *P* > 0.05). Only 0.048% ± 0.033% of the cells remained viable after 24 h, which is similar to the fraction of persister cells reported by others [20]. This fraction of persister cells is too low to study a further decline in viable cells during subsequent screening for novel antibiotics with activity against this population. We therefore proceeded to quantify antibiotic tolerance in stationary-phase cultures, which have been reported to contain a higher fraction of persister cells due to the activation of various stress responses that are associated with persistence [35,37].

The death rate was much slower for stationary-phase cultures at the two highest ciprofloxacin concentrations (at 50× MIC: −0.181 ± 0.018 vs. −0.615 ± 0.061 logCFU۰h^−1^, one-way ANOVA *P* < 0.0001; at 100× MIC: −0.171 ± 0.012 vs. −0.567 ± 0.102 logCFU ۰h^−1^, one-way ANOVA, *P* < 0.0001). However, at 10× MIC, the initial death rate was similar in stationary- and exponential-phase cultures (−0.684 ± 0.069 vs. −0.695 ± 0.138 logCFU۰h^−1^, one-way ANOVA, *P* = 0.8426), and this sample was the only stationary-phase culture that displayed a biphasic time-kill curve (Fig. 2b). The fast initial death rate at the lowest ciprofloxacin concentration could indicate activation of prophages in the genome [22,38]. Earlier studies have shown that low antibiotic concentrations lead to prophage activation, and thereby, a higher killing rate compared to higher antibiotic concentrations. It is therefore important to use antibiotic concentrations that are high enough to avoid this effect.

The median fraction and range of stationary-phase cells surviving 24 h treatment at all ciprofloxacin concentrations were 0.0037% ± [0.0011,0.0153], which, unexpectedly, were lower than for exponential-phase cells (Mann–Whitney *U*-test, *P* < 0.0001). What would have appeared as increased tolerance in stationary-phase cultures sampled after 3 or 5 h antibiotic exposure was thus reversed after 24 h exposure.

It is important to note that the death rate of cells transferred from stationary-phase cultures to TSB with antibiotics reflects both the antimicrobial action of the drug on non-growing stationary-phase cells and also the antimicrobial action on cells that resume growth and lose the tolerant phenotype as nutrients become available. The death rate observed for stationary-phase cultures may simply reflect the rate at which bacteria resume activity after transfer to TSB rather than the rate at which ciprofloxacin kills non-growing cells. The rate with which bacteria resume activity after transfer from nutrient-poor to nutrient-rich conditions depends on the mechanisms the cells use to resume metabolic activity and protein synthesis. For example, Gram-negative *E. coli* must reactivate dimerized ribosomes before protein synthesis can resume and cells revert from the persister phenotype to resume growth [34]. In *S. aureus*, dimerized ribosomes do not disassemble easily upon transfer to fresh media, and in contrast to *E. coli*, ribosome dimerization occurs in all growth phases [39].

Cells in the stationary phase quickly resume growth after transfer to fresh media, and inducing a non-growing state through other means than a lack of nutrients could perhaps delay or slow down the rate of reactivation after transfer to TSB. This has been achieved in *E. coli* using a short exposure to a high concentration of rifampicin to stop transcription, leading to 59% of the population surviving 3 h of ciprofloxacin treatment and being defined as persister cells [20]. We therefore proceeded to determine if the same could be achieved for *S. aureus*. Rifampicin pre-treatment for 30 min induced a non-dividing state, which lasted at least 7 h after transferring the culture to TSB (Fig. 2c). There was an initial loss (~70%) of cells, but all the surviving cells displayed the persister phenotype and tolerated ciprofloxacin for at least 7 h, corroborating results obtained with *E. coli* [20,24]. At 10 h incubation, the time-kill curves of the ciprofloxacin-treated cultures started to diverge from the untreated controls, indicating that cells were resuming activity and becoming susceptible. After 24 h incubation, the untreated cultures were proliferating, while in the ciprofloxacin-treated cultures, only 0.79% ± 0.20% of cells had survived. In conclusion, a short rifampicin pre-treatment generates a high concentration of persister cells, but the persister state only lasts a few hours.

### Continued starvation keeps the *S. aureus* population in a persister phenotype

Assays used to screen compounds for biocidal activity against persister cells should use a culture with a high concentration of persister cells, such that a 1000-fold reduction in viable cells can be detected. Furthermore, the fraction of tolerant cells should remain constant during the incubation. To enable longer incubation times, we investigated if bacteria could simply be kept in a starved state during antibiotic exposure by transferring them to a carbon-free minimal medium.

**Fig. 3.**
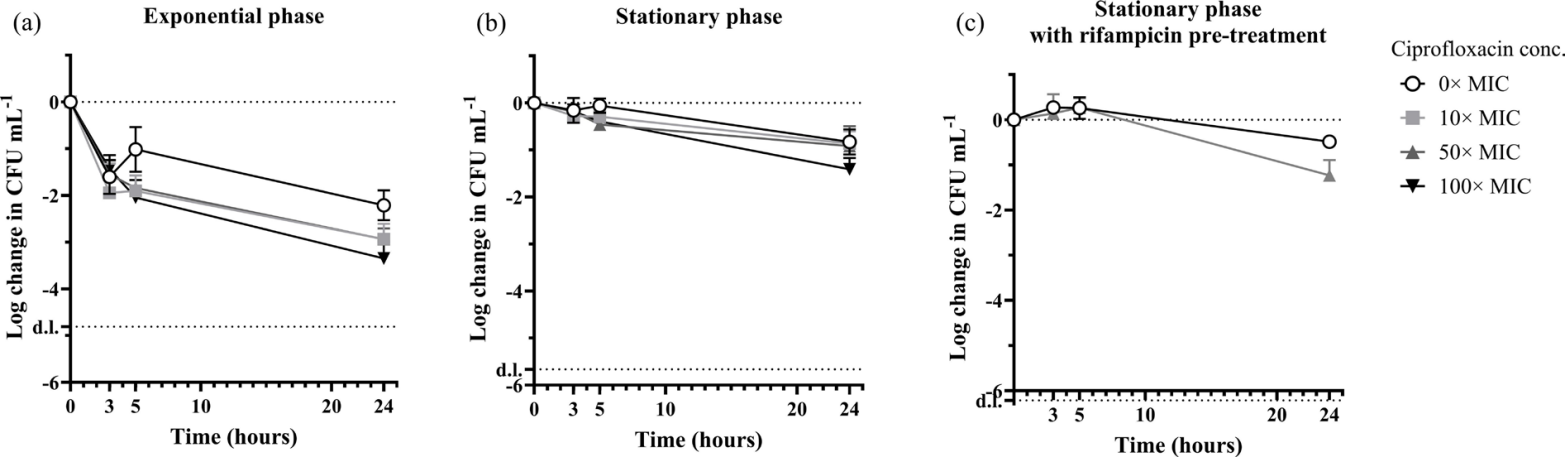
Time-kill curves of *S. aureus* in mM9 buffer. An exponential culture (approximately 5×10^6^ CFU ml^−1^) (a) or a stationary culture (approximately 5×10^7^ CFU ml^−1^) (b) grown in TSB was transferred to mM9 buffer with or without ciprofloxacin at 0 h (1× MIC = 1 µg ml^−1^). (c) A stationary culture of *S. aureus* (approximately 5×10^7^ CFU ml^−1^) grown in TSB was transferred to mM9 buffer +100 µg ml^−1^ rifampicin for 30 min and subsequently washed and transferred to mM9 buffer with or without ciprofloxacin at *T* = 0 h. d.l.: detection limit, *n* ≥ 3 biological replicates.

The exponential-phase culture transferred displayed an initial loss of CFU after transfer to the carbon-free medium (Fig. 3a). This is most likely due to the abrupt transfer from a fast-growing state to a nutrient-free medium. The CFU concentration in the control then increased between the 3 and 5 h sampling points, indicating that some cells had recovered from the transfer and could be detected as CFU The death rate from 5 to 24 h was the same for all samples, including the untreated control (one-way ANOVA, *P* > 0.05), indicating that ciprofloxacin had no effect on the cells in this state. While only 0.11% ± 0.10% of the population survived 24 h exposure to ciprofloxacin, this number corresponds to 30.2% ± 11.9% of CFU in the untreated sample. We therefore conclude that this approach generates a high fraction of antibiotic-tolerant cells but with a high ‘background’ death rate caused by starvation.

We hypothesized that stationary-phase cells would be better equipped to handle the transition to a carbon-free medium, as metabolic processes have slowed down and mechanisms to cope with starvation have been activated. We therefore repeated the experiment with stationary-phase cultures (Fig. 3b). In this case, the death rate was lower than in the untreated control. Ciprofloxacin had no effect on cell viability at 3, 5 and 24 h sampling points when treated with 10× or 50× MIC (Mann–Whitney *U*-test, *P* > 0.05), and only a small effect was seen after 24 h at 100× MIC (*P* = 0.0286) (Fig. 3b). This result indicates that all cells in the population were tolerant of antibiotics. The time-kill curve was not biphasic, and this was expected because it did not consist of two sub-populations with high and low antibiotic tolerance.

Although starvation provided the antibiotic tolerance we had aimed for, we also investigated if the persister-inducing treatment with rifampicin prior to incubation with ciprofloxacin affected the overall survival and tolerance to ciprofloxacin under these conditions. Again, we observed no effect of ciprofloxacin on CFU after 3 or 5 h incubation (3 h: *t*-test, *P* = 0.6242, 5 h: Mann–Whitney *U*-test, *P* > 0.9999) (Fig. 3c), while a small effect was detected after 24 h at 50× MIC (*t*-test, *P* = 0.0086). We therefore chose to proceed with the assay using a stationary culture transferred to mM9 without pre-treating with rifampicin.

### Continued starvation only keeps a small fraction of *E. coli* in a persister phenotype

Due to the large body of work on the persister phenotype in *E. coli*, we wanted to do a side-by-side comparison of *E. coli* and *S. aureus*. Stationary-phase cultures were divided into two groups. One group received 30 min rifampicin treatment to induce the persister phenotype, while the other group did not. All samples were then exposed to 50× MIC ciprofloxacin in growth media (TSB) or under starvation in carbon-free minimal media (mM9) for 24 h.

**Fig. 4.**
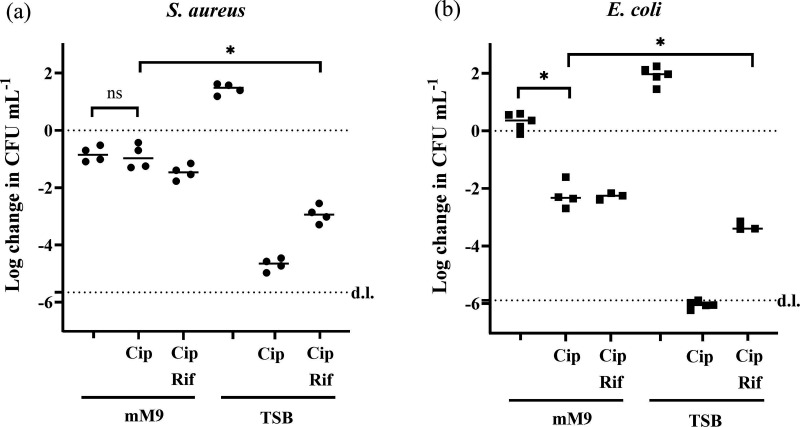
Change in log CFU ml^−1^ after 24 h treatment. *S. aureus* (a) or *E. coli* (b) were grown in TSB overnight and transferred (approximately 5×10^7^ CFU ml^−1^) to either TSB or mM9 buffer with or without 50× MIC ciprofloxacin (Cip) (for *S. aureus* 1× MIC = 1 µg ml^−1^, for *E. coli* 1× MIC = 8 ng ml^−1^). Samples received in 30 min. Then, 100 µg ml^−1^ rifampicin pre-treatment prior to ciprofloxacin treatment was included (Rif). *P < 0.0001, ns: *P* > 0.05, one-way ANOVA, *n* ≥ 3 biological replicates.

As expected, ciprofloxacin treatment in growth media resulted in very low survival rates in both *S. aureus* and *E. coli* (0.002% ± 0.001% and 0.0009% ± 0.0003%, respectively) (Fig. 4). Under these conditions, the stationary-phase cultures resume growth and become susceptible to the antibiotic. Pre-treatment with rifampicin did increase the fraction of tolerant cells by several orders of magnitude, but it did not protect the entire population. This was expected, as rifampicin’s effect on antibiotic tolerance did not last for the full 24-hour incubation period, and cells would resume activity and become susceptible after some hours (Fig. 3c). In contrast, ciprofloxacin treatment in a carbon-free minimal medium resulted in much higher survival rates: 86.0% ± 31.8% for *S. aureus* and 0.29% ± 0.27% for *E. coli* (Fig. 4). Pre-treatment with rifampicin did not improve antibiotic tolerance under these conditions (one-way ANOVA, *P* = 0.8816).

We, therefore, conclude that controlled starvation obtained by transfer of stationary-phase cells to a carbon-free minimal medium can be used to generate a high concentration of bacteria, which display the antibiotic tolerance of the persister phenotype during 24 h incubation with antibiotics. This method is thus suitable for preparing bacteria for high-throughput screening to discover antimicrobials that target non-growing, antibiotic-tolerant bacteria. If the incubation period with antibiotics is shorter (<7 h), a short pre-treatment with rifampicin prior to antibiotic exposure in growth media is also a powerful way of inducing transient antibiotic tolerance, which was shown for *E. coli* previously [20] and for *S. aureus* in this study (Fig. 3c).

There is much discussion in the field about what characterizes persister cells, e.g. whether persister cells are truly dormant or retain some activity in order to avoid killing by antibiotics [3,21, 40]. Here, we generate tolerant *S. aureus* that survives 24 h of 50× MIC ciprofloxacin by transferring stationary-phase cells to a minimal medium where they cannot increase metabolic activity or resume growth. It was previously reported that to generate true *E. coli* persister cells using starvation, the cells must experience starvation for 7 weeks [25,41]. One could thus argue that starved *S. aureus* are not persister cells but merely resting cells that are not dividing due to lack of nutrients but otherwise maintain some level of activity that enables fast reactivation after transfer to a nutrient-rich medium. This is likely the case. However, for their application in the discovery of novel antibiotics to treat biofilm infections, the key questions are (1) whether these cells represent the antibiotic-tolerant phenotype of cells in biofilm infections and (2) whether antimicrobials that display activity against these starved cells will also display the same activity against biofilm infections. The answers to both questions remain unknown. Nevertheless, we show that starved cells display the same tolerance to ciprofloxacin and susceptibility to mitomycin C, as others have described for persister cells and biofilms [29,42]. Importantly, the antibiotic tolerance was sustained during the 24 h incubation, and we could detect more than a 100 000-fold reduction in viable cells when treated with mitomycin C (Table 1), which makes the assay highly sensitive. In our subsequent quest to discover molecule structures with antimicrobial activity against *S. aureus* persister cells, we chose to incubate cells in a carbon-free minimal medium to select compounds based on their direct antimicrobial activity on non-growing cells over 24 h.

**Table 1.** Log reduction in viable *S. aureus* (Δlog) in response to treatment

Compound	1 mM	5 mM	10 mM
**258**	≥5	≥5	≥5
**260**	≤2	≥5	≥5
**267**	Between 2 and 4	Between 2 and 4	Between 2 and 4
**280**	Between 2 and 4	≥5	≥5
**292**	≤2	Between 2 and 4	Between 2 and 4
**296**	Between 2 and 4	Between 2 and 4	Between 2 and 4
**322**	Between 2 and 4	≥5	≥5
	**10×MIC**	**50×MIC**	**100×MIC**
**Ciprofloxacin**	≤2	≤2	≤2
**Mitomycin C**	≥5	≥5	na

*S. aureus* persister cells were treated with numbered compounds at different concentrations. After 24 h treatment, 10 µl of each undiluted sample was spotted on an agar plate and incubated overnight. The survivors were semi-quantified from the agar plates in units of CFU ml−1 and normalized to the CFU ml−1 at the start of the incubation (107). ‘≥5’ denotes full inhibition (no colonies), as the detection limit of the assay is ≤102 CFU ml−1. ‘≤2’ denotes no detectable inhibition. For any sample where there is inhibition but the colonies are non-discernible, the log change of survivors is between 2 and 4. This is based on separate experiments showing that the culture resulted in a non-confluent layer of bacteria when at 105 CFU ml−1, enabling detection of a 2–4 log reduction in CFU, even when colonies could not be counted. *n* = 3 biological replicates. na: this compound concentration was not tested.

### Screening-tolerant *S. aureus* has the potential to identify novel anti-persister drugs

We started with a broad selection of compounds that were smaller than common antibiotics but had structural similarities to previously published compounds with activity against persister cells [43]. Our strategy was to identify molecular structures in small molecules that have activity, such that these structures can be applied in the design of more potent molecules with several active structures. This approach avoids screening compound libraries with large molecule structures that are unsuited for subsequent drug development.

**Fig. 5.**
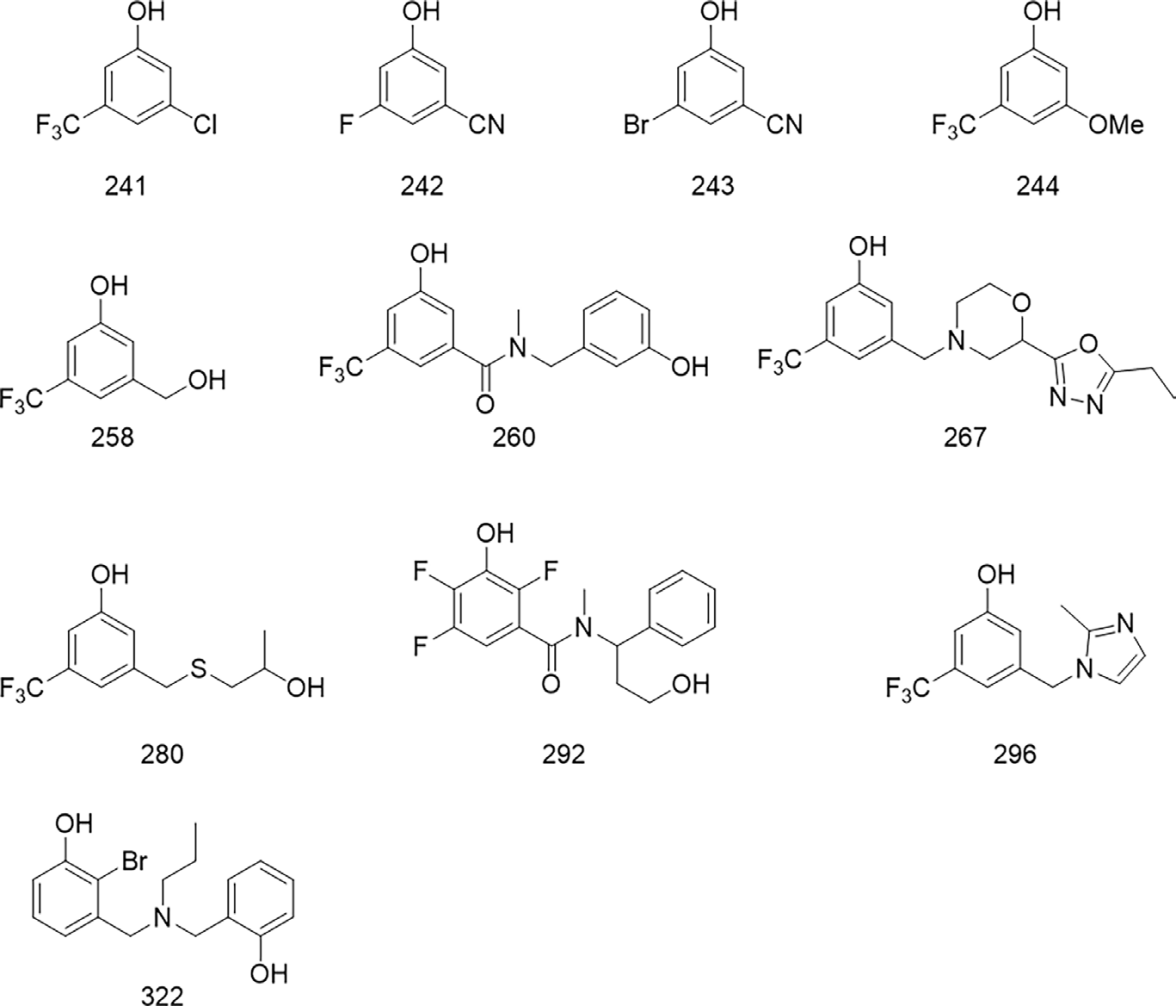
The structures of potential anti-persister compounds tested against starvation-induced tolerant cells. Two hundred and fifty compounds were pre-screened for anti-persister activity, yielding a cluster of four fragments (compounds 241, 242, 243 and 244) with the same 3,5-disubstituted phenol motif. This motif was used to design potential anti-persister compounds that were further screened using the method demonstrated here.

We tested the antimicrobial activity of compounds at 1, 5 and 10 mM, and subsequent rounds of compound design were then based on molecular structures that indicated activity. The antimicrobial efficacy was observed by spotting 10 µl of the bacterial suspension on agar and scoring the activity based on the absence of colonies (CFU ml^−1^ ≤ 10^2^), the presence of discernible colonies (CFU ml^−1^ = 10^2^–10^4^) or a confluent layer of bacteria (CFU ml^−1^ ≥ 10^4^). As the cell concentration in the untreated control was approximately 10^7^ CFU ml^−1^, any effect detected by this assay would indicate a reduction in CFU of at least 2 log. Mitomycin C and ciprofloxacin (at 50× MIC) were included as controls to verify the persister phenotype, which is killed by the former and tolerates the latter (Table 1). Initial screening of 250 compound fragments resulted in a cluster of four compounds having the same 3,5-disubstituted phenol motif (Fig. 5). Commercially available compounds having this or a similar structure to this active fragment were selected for testing. Of these tested compounds, compound 258 (Fig. 5) was identified as the most promising with antimicrobial, activity resulting in >log5 reduction in viable cells at 1 mM (Table 1).

**Fig. 6.**
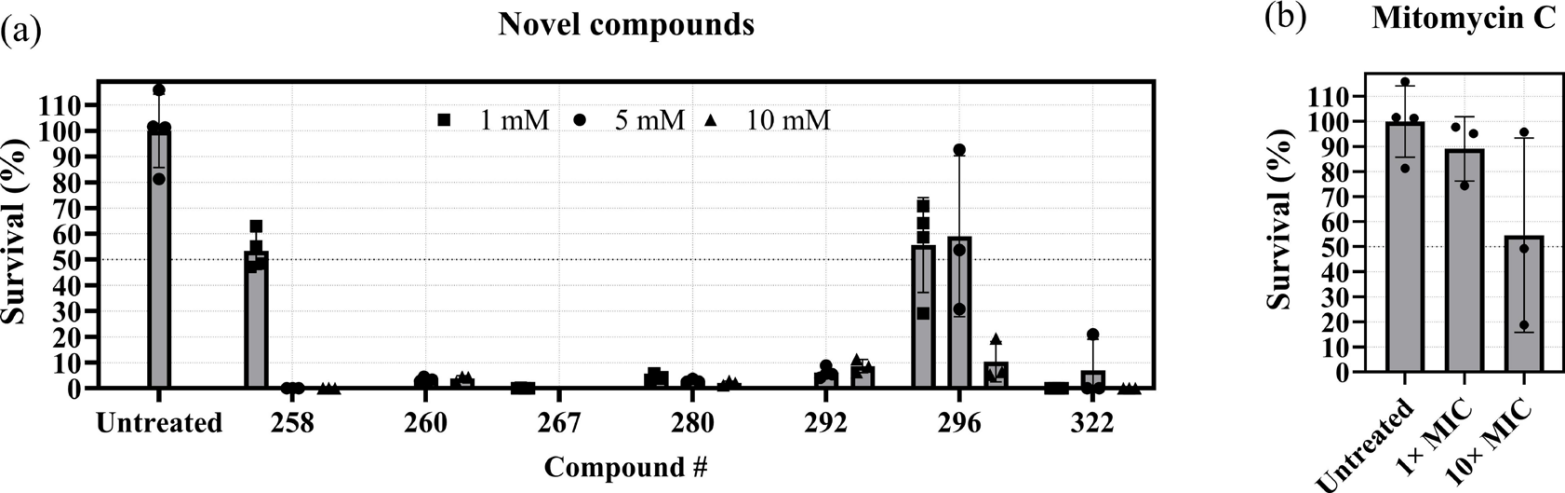
Cytotoxicity of compounds and mitomycin C against CHO cells. CHO cells were grown to 70–90% confluency in Ham’s Nutrient Mixture F12 in 96-well plates and subsequently treated with the specified compounds (a) or mitomycin C (b) for 24 h at 37 °C, 5% CO_2_ and 85 % humidity. Subsequently, CHO cells were treated with 1× PrestoBlue and incubated for 2 h at 37 °C, 5% CO_2_ and 85 % humidity before reading the plate in a plate reader at 560 nm excitation and 590 nm emission. *n* = 3–4 biological replicates. The survival of each sample was normalized to the average of the corresponding solvent control.

To determine whether this activity is specific to bacteria, we performed a cell viability assay against a rodent cell line. The cell line was treated with compounds at similar concentrations as the persister cells and was likewise treated for 24 h before cell viability was assessed. Compounds 258 and 296 showed approximately 50% reduction in viability at 1 mM and at 1 mM and 5 mM, respectively (Fig. 6a). There is thus considerable general cytotoxicity, although we saw a much larger reduction in bacterial viability. For the remainder of the compounds, we observed the highest cytotoxicity, with an average cell viability below 10% (Fig. 6a). We compared the cytotoxicity of the compounds with the cytotoxicity of mitomycin C as a control. At 10× MIC mitomycin C, we saw approximately 50% cell line viability, which is similar to the observed cytotoxicity at 1 mM compound 258 and at 1 mM and 5 mM compound 296 (Fig. 6b). Furthermore, at 1 mM, the antimicrobial activity of compound 258 was similar to that of 10× MIC mitomycin C. The high cytotoxicity may be the cost of the efficiency of these compounds. Further screening is therefore needed to identify motifs with lower general cytotoxicity and higher specificity against bacteria.

### Strategies for high-throughput screening to identify anti-persister drugs

The key to high-throughput screening is scalability and automatization. In our assay, we assessed bacterial viability by scoring CFU on agar without prior dilution. An even simpler approach would be to transfer the 10 µl to TSB in a new microwell plate and score the outcome based on the presence/absence of viable cells that would lead to growth measured as optical density during a subsequent incubation. This transfer and readout can be fully automated with common robotic systems, but it would also narrow the read-out to a yes/no answer to whether any viable cells remained after the incubation.

Other high-throughput assays have assessed viability based on loss of membrane permeability using membrane-impermeable fluorescent DNA-binding dyes. Using a fluorescent readout also enables automated analysis of the result in a microwell plate format, but the approach will be biased towards identifying membrane-active compounds or compounds that lead to cell lysis through other means. A highly effective drug such as mitomycin C would be missed in this assay, as it kills cells by crosslinking DNA and does not immediately lead to cell lysis (data not shown). The approach was applied to an 85 000 compound library, leading to the identification of compound NH125, a bacterial histidine kinase inhibitor with anti-persister activity [44]. The same group used a methicillin-resistant *S. aureus* (MRSA) killing screening method in the nematode *Caenorhabditis elegans* to screen 82 000 compounds and identified a new class of synthetic retinoids that are effective against MRSA persister cells [45].

Some anti-persister compounds kill persister cells together with conventional antibiotics by ‘waking up’ the persister cells, thereby disarming defence mechanisms [46,48]. Screening for compounds with this effect must occur in a nutrient-rich medium and in combination with other antibiotics in order to identify the killing propensity of the combination. Otherwise, the compounds should be screened based on persister resuscitation and not persister killing, which likewise has been shown to be a successful approach [48]. Our assay is therefore not suited to identify this type of anti-persister drug, but our results indicate that such drugs could be identified by inducing the persister phenotype through rifampicin exposure and subsequently testing the antimicrobial effect in TSB with ciprofloxacin during <5 h incubation.

One of the newest trends in compound screening is the use of machine learning with training and predictions to initiate a screening process. Stokes *et al*. used deep learning in antibiotic discovery, where they used a training set of 10^4^ compounds to initiate a baseline for a successful compound [49]. Subsequently, they carried out a large-scale prediction of 10^8^ small molecules in a chemical landscape. When they narrowed the compounds down to 10^5^–10^6^ compounds, they used a conventional molecule screening method for validating the hits by incubating *E. coli* in nutrient-rich media containing the selected compounds to determine growth inhibition. This approach led to the discovery of halicin, a molecule that is structurally divergent from conventional antibiotics and displays bactericidal activity against active cells as well as inactive persister cells. The anti-persister activity of halicin was perhaps a stroke of luck since the discovery process was not targeted towards anti-persister compounds. However, the use of machine learning to identify bactericidal (rather than bacteriostatic) drugs increases the chance of finding antibiotics that kill persister cells. Machine learning can be very efficient when initializing a new screening of bactericidal drugs. However, it must always be followed with experimental evidence, and high-throughput assays that reliably detect anti-persister activity with high sensitivity are thus needed to aid the discovery of novel antibiotics to treat recalcitrant bacterial infections harbouring persister cells. With our method, there is potential to discover new anti-persister drugs by screening cells that are highly tolerant to conventional antibiotics.

## Abbreviations

CCCP: carbonyl cyanide *m*-chlorophenylhydrazone
*E. coli*: *Escherichia coli*
mM9: modified M9
MRSA: methicillin-resistant *S. aureus*
*S. aureus*: *Staphylococcus aureus*
TSA: tryptic soy agar
TSB: tryptic soy broth
